# Knowledge Structure and Evolution of Wetland Plant Diversity Research: Visual Exploration Based on CiteSpace

**DOI:** 10.3390/biology14070781

**Published:** 2025-06-27

**Authors:** Xuanrui Zhang, Shikun Chen, Pengfu Yao, Jiahui Han, Ri Jin

**Affiliations:** 1 College of Geography and Ocean Sciences, Yanbian University, Hunchun 133300, China; 2224273498@ybu.edu.cn (X.Z.); 2224273499@ybu.edu.cn (S.C.); 2 Department of Physical Geography and Ecosystem Science, Lund University, SE-223 62 Lund, Sweden; fudisanri@gmail.com; 3 Songpan Ecological Environment Bureau, Aba Tibetan and Qiang Autonomous Prefecture, Sichuan Province, Songpan 623300, China; hjhmaz@163.com

**Keywords:** wetland plant diversity, habitat conservation, biodiversity, CiteSpace, vegetation

## Abstract

Plant diversity, as a critical indicator of wetland ecosystem health and functionality, has garnered extensive research attention. However, systematic and quantitative assessments of research advancements in wetland plant diversity remain inadequate. This study pioneers a global bibliometric analysis of wetland plant diversity research (1986–2025), designed to systematically examine its worldwide patterns, knowledge architecture, and evolutionary trends. Bibliometric analysis was performed using CiteSpace V6.2.R4 (64-bit) software on 482 publications retrieved from the Web of Science Core Collection. Results indicate that the United States, Canada, China, and several European countries have collectively prioritized wetland plant diversity research, forming a close international collaboration network. Research themes initially centered on species composition, community structure, and diversity metrics have expanded to multiple dimensions such as ecosystem functions and services, environmental change impacts, and wetland management and restoration, forming several key research clusters. Keyword time-zone mapping reveals the trajectory of research themes from basic descriptions to applied and environmental relevance, while emergent analyses accurately identify hotspots and frontiers of current research such as ecosystem services, functional diversity, and climate change impacts. These findings contribute to comprehending the overall framework and developmental trajectories in wetland plant diversity research, and provide a reference for identifying potential research gaps and planning future research directions.

## Simple Summary

Wetlands are crucial ecosystems that provide clean water, flood control, and wildlife habitats, but they face serious threats from human activities and climate change. Since plants are fundamental to wetland health, understanding how scientists study plant diversity helps improve conservation efforts. This research analyzed patterns in 482 scientific publications (1986–2025) to map global progress in wetland plant studies. The key findings show that research has grown significantly, led by countries like the United States, China, and European nations working together. Early studies focused on counting plant species, but recent work explores how climate change and human impacts affect wetlands, using technologies like satellite monitoring. The results highlight urgent priorities: protecting wetlands from pollution and habitat loss, restoring damaged areas, and preparing for climate shifts like sea-level rise. These insights will help governments and conservation groups make smarter decisions to safeguard wetlands—ensuring they continue supporting clean water, wildlife, and community resilience worldwide.

## 1. Introduction

Wetlands account for about 6% of the world’s total area and are an important ecosystem on the earth. They play a key role in water conservation, flood regulation and storage, water quality purification, biodiversity maintenance and carbon storage [1,2]. However, in recent years, wetlands have been affected by human activities and climate change, and wetland ecosystems have become one of the most threatened ecosystems in the world [3]. As the basis of its structure and function, wetland plant diversity is the core indicator to measure the health and ecological service value of wetlands [4], which directly affects the primary productivity, nutrient cycling and habitat provision capacity of wetlands [5,6]. Therefore, it is important to understand the spatiotemporal patterns, influencing factors and response mechanisms of wetland plant diversity for wetland conservation, management and restoration [7,8].

Wetland plant diversity research has attracted widespread attention globally and has shown interdisciplinary and diversified characteristics [9,10]. Scholars have adopted a combination of methods, such as traditional field investigations and field experimentation, to obtain data on community composition, richness, biomass, etc. [11], laying the foundation for a deep understanding of the plant diversity characteristics of specific areas; Concurrently, remote sensing-based approaches are being implemented to assess macroscale vegetation diversity patterns [2,12], demonstrating significant potential for rapid, large-area wetland monitoring. In addition, controlled experiments have been used to explore the relationship between diversity and ecosystem functioning, such as Lisa D. Williams et al. [13], who control initial planting richness and environmental conditions in the middle universe to determine the role of initial planting richness in vegetation community development. Statistical modeling frameworks are being extensively applied for both assessment and predictive modeling of wetland vegetation diversity [14,15]. For example, Rachel Schultz’s team [16] employed NMDS to delineate phytocommunity structures, subsequently developing structural equation models to quantify interactions between diversity indices, community parameters, and carbon cycling processes. Wang et al. [17] utilized Shannon–Wiener and Simpson indices to evaluate wetland vegetation restoration process in Northeast China’s degraded ecosystems. Syntheses of these methodological advances have yielded critical insights: hydrological regimes have been identified as principal determinants of wetland plant community organization [18]; moderate disturbance is usually beneficial to maintaining high species diversity [19]. Anthropogenic impacts—including habitat alteration, invasive species proliferation, and agrochemical contamination (e.g., herbicides)—are becoming more and more prominent, posing a serious threat to the diversity of wetland plants and even leading to the loss of biodiversity [20,21,22]. The imperative for longitudinal monitoring programs has been emphasized to track temporal biodiversity trends [23].

Bibliometric methodologies leverage citation networks and keyword co-occurrence patterns to quantitatively delineate research fronts, intellectual foundations, collaborative clusters, and developmental trajectories within specialized domains, thereby elucidating the epistemological architecture and cutting-edge directions of knowledge fields [24]. Recent cross-domain applications validate its efficacy in detecting paradigm shifts, as exemplified by Yang et al. [25] used CiteSpace to present a holistic picture of ecosystem health development and emphasized the prospects for large-scale ecosystem assessment based on remote sensing, while Wu et al. [26] comprehensively analyzed the processes and mechanisms of carbon emissions from agroecosystems by reviewing the literature over the past 30 years. Currently, review research in this field often has some limitations and its interdisciplinary characteristics are ignored. For example, Sun et al.’s [2] focused only on the research trend of remote sensing technology in monitoring vegetation diversity in wetlands at large scales, while neglecting climate-vegetation interactions and biodiversity-carbon nexus. In contrast, bibliometric analysis proves particularly valuable for wetland phytodiversity research, where rapid technological convergence and multidisciplinary intersections have generated fragmented knowledge landscapes necessitating macro-scale quantitative mapping to systematically present the overall structure and evolutionary trends of the field. Therefore, this study aimed to solve the key gaps by using CiteSpace software to perform a quantitative analysis of the wetland plant diversity-related literature: (1) to reveal the evolution of wetland plant diversity research since 1986 through citation network mining; (2) identification of research clusters and frontiers through keyword co-occurrence networks and timeline visualization; (3) synergistic integration of temporal trend analysis with technological forecasting to anticipate next-generation methodological paradigms. The resulting framework aims to systematize the scattered knowledge of wetland plant diversity through bibliometric analysis, provide a scientific basis for subsequent researchers to quickly grasp the dynamics of the field, find research entry points, and provide more comprehensive information support for the conservation and management of wetland plant diversity.

## 2. Data and Methodology

### 2.1. Data Collection and Search Strategies

The bibliographic dataset for this investigation was retrieved from the Web of Science Core Collection, a premier citation index curated by Clarivate Analytics. The selection of WoS Core Collection as the primary data source was predicated on its comprehensive coverage of high-impact scholarly journals and complete citation metadata, establishing it as the authoritative repository for bibliometric studies requiring citation network visualization and knowledge mapping [27]. Since WOS records show that the first manuscript on “wetland plant diversity” was published in 1986 [28], we limited the scope of the study to 1986 to 2025. To ensure retrieval completeness and precision, the search strategy incorporated controlled vocabulary and natural language terms related to “wetland” and “plant diversity”, with the Boolean search query being iteratively refined through scope validation protocols as follows:

(TI = (“wetland” OR “marsh” OR “swamp” OR “bog” OR “fen” OR “peatland” OR “mangrove”) OR AB = (“wetland” OR “marsh” OR “swamp” OR “bog” OR “fen” OR “peatland” OR “mangrove”)) AND TS = (“plant diversity” OR “vegetation diversity” OR “flora diversity”) AND TS = (“species richness” OR “functional diversity” OR “community structure” OR “species diversity” OR “biodiversity”)

Duplicates were removed through CiteSpace’s built-in deduplicator. Subsequently, three independent reviewers applied inclusion/exclusion criteria to assess title/abstract relevance: (1) document type: articles/reviews; (2) language: English-only; (3) discipline screening: ecology, environmental sciences, plant sciences, etc.; (4) exclusion: non-relevant domains (e.g., immunology, energy fuels). After manual screening and deduplication, 482 valid articles related to wetland plant diversity were obtained, which were then exported in “RefWorks” plain text format with complete metadata.

### 2.2. Analytical Methods

This investigation employs bibliometric methodologies with CiteSpace V6.2.R4 (64-bit) Advanced serving as the primary analytical and visualization platform. Developed in Java by Chaomei Chen at Drexel University (2004), CiteSpace is an information visualization tool specializing in mapping epistemic architectures, research frontiers, and evolutionary trajectories within the scientific literature, with particular efficacy in detecting pivotal nodes (publications, authors, institutions) and intellectual turning points [29]. The software then forms these nodes into an interconnected network, with node sizes and color intensities showing the influence relationships between documents [30]. The node size indicates the frequency of occurrence, while the node color indicates the year. Its core capability lies in processing extensive bibliographic corpora to uncover latent knowledge structures, dynamic evolutionary patterns, and emerging research fronts, thereby providing panoramic insights into wetland phytodiversity studies.

The main steps are as follows: First, create a new CiteSpace project and import the complete records and reference data described above into it. Second, configure the relevant parameters in the project, and set Time Slicing to one year interval, that is, separate analysis and consolidation every year. Subsequently, different nodes are selected according to the content of the analysis: the node type is set to “Author” and “Institution” to identify the core author group, the main research institutions and their partner networks; the node type is set as “Country” to analyze the cooperation intensity and cooperation mode of different countries or regions in the field of wetland plant diversity research; the node type is set to “Cited Reference” to identify the knowledge base, core literature, and research paradigm of the field; the node type is set to “Keyword” to draw a keyword co-occurrence network map, identify research hotspots, and detect research frontier topics that have significantly increased attention in a specific time period. The parameters for each analysis are given in the upper left corner of the corresponding image. This multi-layered analytical framework systematically elucidates the epistemological architecture, evolutionary trajectories, and prospective developments in wetland phytodiversity research. Finally, by comparing these visual representations longitudinally and horizontally, the node size, link density and key salient features in the visualization are examined in detail, aiming to systematically and objectively reveal the knowledge structure, evolutionary path and future development trend in the field of wetland plant diversity research, so as to provide valuable support for further research and theoretical expansion.

## 3. Results and Discussion

### 3.1. Publications and Annual Growth

**Figure 1:**
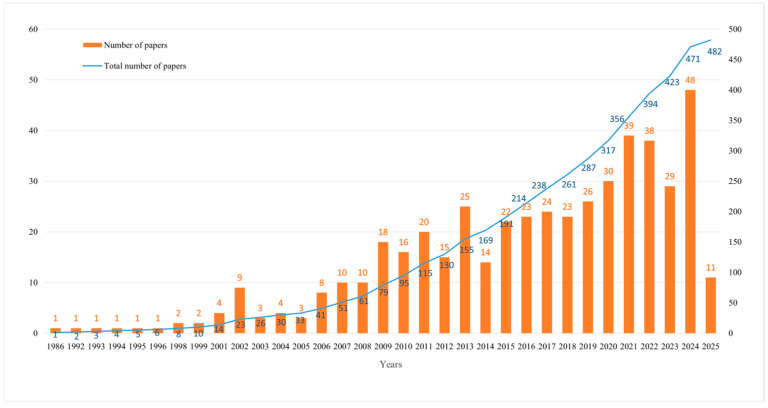
Number of articles published per year from 1986 to 2025.

Figure 1 depicts three different stages in the evolution of wetland plant diversity research, quantified by a bibliometric analysis of 482 publications (1986–2025):

The first phase is Nascent Exploration (1986–2006): an average of <10 papers per year, with extremely limited publications reflecting the emerging state of the field. The basic work during this period is to explore the impact of various factors on wetland plant diversity, such as disturbance; physical resources, etc. [31,32]. Building upon these efforts, Xiong et al. [33] took the lead in exploring the impact of different influencing factors (elevation, vegetation canopy, litter and seed availability) on wetland plant diversity through factorial experiment. The results show that some individual factors may have little impact, but their interactions may have a significant impact on overall species diversity and plant colonization. The interaction effect explains 41–63% of the total change in species richness and the number of individuals grown from added seeds, laying the foundation for future ecological modeling and ecosystem restoration research. Concurrently, scientific attention began shifting toward understanding functional linkages between floristic diversity and wetland ecosystem services [34].

The second phase is Methodological Expansion (2007–2019): the increase in publications is more pronounced and this growth is closely linked to advances in biodiversity survey techniques such as remote sensing and molecular ecology methods. For example, Shackleton et al. [35] used environmental DNA to assess the spatial composition of wetland vegetation communities, resulting in derivative landscape patterns of plant communities. Intensified focus on wetland ecosystem services—particularly carbon sequestration—functioned as a key growth catalyst during this period [36]. During this period, researchers delved into and developed a large number of ecological models to deal with different specific wetland types [15,37]. For example, Wingard et al. [38] developed an integrated conceptual ecological model (ICEM) for coastal wetlands in southwestern Florida that demonstrates the links between drivers, stress, ecological processes, and ecosystem services. Zhao et al. [37] used the system dynamics software STELLA V1.1 to construct the Hanshiqiao wetland ecological model based on nutrient circulation in the food network, and conducted a detailed comparative analysis of the aquatic ecological ecological status of Hanshiqiao wetlands to evaluate the impact of ecological restoration on Hanshiqiao wetlands.

The third stage is technology convergence (2020–2025): it is worth noting that the field has entered a phase of high growth. By 2023, cumulative publications surpassed 400, evidencing wetland phytodiversity’s emergence as a mature yet dynamic research domain. This phenomenon and wetland biodiversity research have been integrated into the global sustainable development framework [39]. At the same time, the intensification of the impact of climate change has driven the urgent need for the scientific community to understand and protect wetland ecosystems [40]. The field has benefited from transdisciplinary approaches integrating ecological, hydrological, remote sensing, and socioeconomic methodologies, broadening investigative scope and scientific engagement [9,41]. Overall, this publication trend clearly reflects the evolution of the field from an early slow exploration phase to today’s multidisciplinary focus and rapid growth, with wetland plant diversity continuing to be a high priority focus for ecological research, especially under the pressure of biodiversity loss and climate change.

### 3.2. Subject Area Distribution

**Figure 2:**
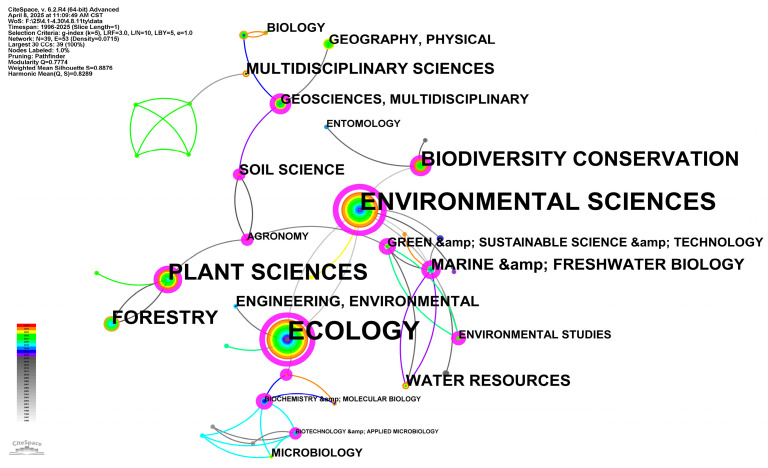
Co-occurrence network diagram for the subject area of wetland plant diversity.

Discipline category-based visualizations show highly modular but closely linked academic landscapes on wetland plant diversity in Web of Science (Figure 2). At its core are ecology, environmental science, and plant science, each of which is represented by large concentric circles, and its colorful layered structure demonstrates the ongoing research activities from the late 1990s to the present. Ecology’s topological prominence (node size/linkage density) underscores its centrality in investigating species richness, functional diversity, and community organization in wetland ecosystems. Environmental science is located next to ecology and serves as a bridge discipline: its link to water resources and green and sustainable science and technology suggests that in wetland biodiversity research, the emphasis on the sustainability of hydrological processes and applications is expanding [18]. Meanwhile, plant science is closely linked to forestry and agronomy, reflecting the widespread attention of wetland plant diversity among different land use types [42].

Secondary clusters encircling the central triad demonstrate the transdisciplinary breadth of wetland phytodiversity research. Biodiversity conservation and water resources form a coherent sub-network that translates ecological insights into conservation planning and hydrological management. Marine and freshwater biology and environmental studies link these clusters, suggesting that researchers are concerned about plant diversity in different water wetland types [43]. At the same time, the adjacency of soil science, geosciences (multidiscipline), and geography underscores growing adoption of pedogeochemical analyses and geospatial modeling to decipher nutrient fluxes, sediment transport regimes, and landscape-scale heterogeneity in wetland plant communities [44,45].

Collectively, these patterns delineate a research domain anchored in classical ecology and botany yet progressively integrated with hydroscience, soil biogeochemistry, conservation biology, and engineering innovations. Higher modularity and key nodes of the network confirm clear thematic clustering, however dense intercluster linking confirms strong interdisciplinary nature (Figure 2). The ascendant trajectory of environmental science and water resources research aligns with global concerns over wetland degradation and water security crises. The emergence of links between ecology and biotechnology and applied microbiology also points to molecular biology and bioengineering methods for evaluating microbial-plant interactions [46]. Future research trajectories will likely strengthen these interdisciplinary nexuses, particularly at the interface of sustainable technologies, molecular tools, and spatial modelling, to address pressing challenges in biodiversity conservation, blue carbon sequestration, and climate-resilient ecosystem services.

The temporal color of the nodes further reveals the focus of the evolution. Early-stage research (gray/light blue) clustered around core ecology-botany-microbiology domains, reflecting studies at the basic taxonomic and community level. Beginning in mid-2005 (green and yellow), the momentum of environmental science and water resources has grown, coinciding with growing global concerns about wetland loss and water security. Recent work (orange to red) has shown a surge in activity in the fields of green and sustainable science and technology, as well as engineering, indicating a shift towards technological solutions for wetland restoration and monitoring.

### 3.3. Core Journals and Literature

**Figure 3:**
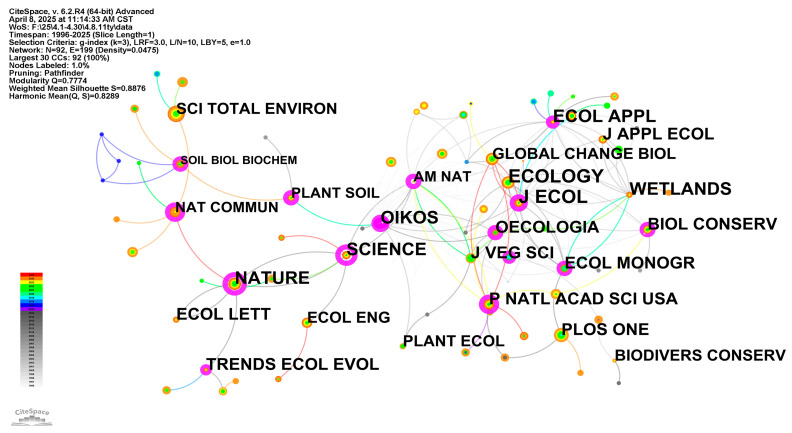
Journal co-citation network of wetland plant diversity research.

Figure 3 displays journals cited at least 100 times, thus forming a co-citation network of journals for wetland plant diversity research. It is evident that the co-citation network reveals core positions occupied by canonical ecology journals—Cology, Journal of Ecology, Oecologia, Oikos and Journal of Vegetation Science—whose large, multicolored nodes demonstrate enduring influence and continued citation in recent years. Surrounding this core, Global Change Biology, Ecology and Applied Ecology and Journal of Applied Ecology serve as pivotal knowledge bridges connecting fundamental ecological theory to biodiversity applications under global change. The proximity between wetlands and biological conservation highlights the translation of ecological insights into protection policies and habitat management, while Plant and Soil, Soil Biology & Biochemistry, and Ecological Engineering form a secondary cluster focused on publishing articles on technical methods for determining the ecological functions of soils and wetlands. High-impact comprehensive journal—including Nature, Science, and PNAS—bolster broader visibility for wetland diversity research, frequently co-cited with thematic journals like Trends in Ecology & Evolution. Over time, the color palette of node colors has shifted from foundational articles in core ecology and plant science to more recent outbursts of activity in sustainability-oriented publications (e.g., Green & Sustainable Science & Technology, PNAS, PLOS ONE), signaling the field’s growing embrace of interdisciplinarity and methodological innovation.

Table 1 presents the top 10 most-cited articles retrieved through CiteSpace (6.2.R4) and Web of Science search functionalities. These articles comprise both research articles and review papers, demonstrating substantial scholarly impact globally. The review articles focus on wetland status quo, ecosystem functions, and imminent threats. The most frequently cited work is Zedler et al.’s (2005) review [4] “Wetland resource: Status, trends, ecosystem services, and restorability” with 1476 citations. This work systematically synthesizes global wetland conditions and their biodiversity maintenance, water purification, flood mitigation, and carbon sequestration capacities, while advocating adaptive management approaches to test alternative restoration techniques in large-scale practical restoration sites. The second most-cited publication is Simon et al.’s (2009) study [47] “Recent assembly of the Cerrado, a neotropical plant diversity hotspot, by in situ evolution of adaptations to fire” with 771 citations. Employing phylogenetic analysis, the team investigated plant diversity in South America’s Cerrado biome, with particular emphasis on both the formation history of species-rich communities and the macroevolutionary processes generating global biodiversity patterns. It also signifies an emerging research focus in wetland ecology on the causal mechanisms underlying plant diversity patterns. The third most-cited work is Brinson et al.’s (2002) review [48] “Temperate freshwater wetlands: types, status, and threats” with 412 citations. This review provides a systematic synthesis of temperate freshwater wetlands, demonstrating that biodiversity loss stems from both habitat area reduction and environmental degradation.

Synthetically, wetland phytodiversity research is rooted in classical ecology and plant science, while progressively expanding into applied ecology, global change, biodiversity conservation, soil science, and ecological engineering. Highly-cited reviews underscore sustained scholarly attention to wetland status, functionalities, and threats, whereas evolutionary mechanism investigations signify the discipline’s probing into diversity origins. Temporally, citation dynamics have shifted from the core foundational literature toward sustainability-oriented, interdisciplinary, and methodologically innovative publications, reflecting the field’s broadening scope and heightened emphasis on practical solutions.

### 3.4. Core Authors

**Figure 4:**
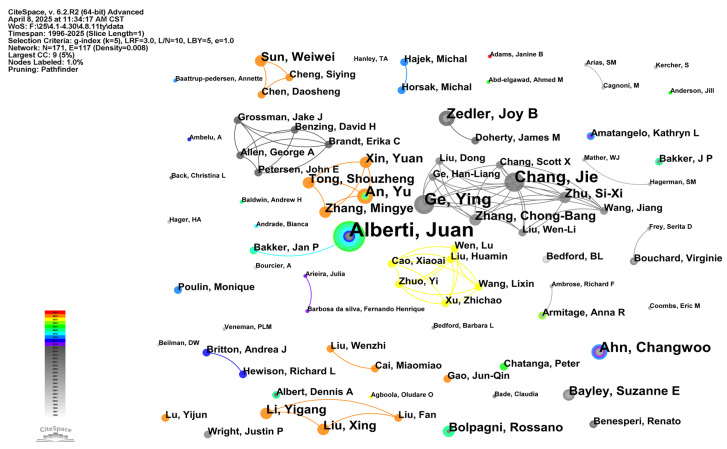
Author co-occurrence map of wetland plant diversity research.

**Figure 5:**
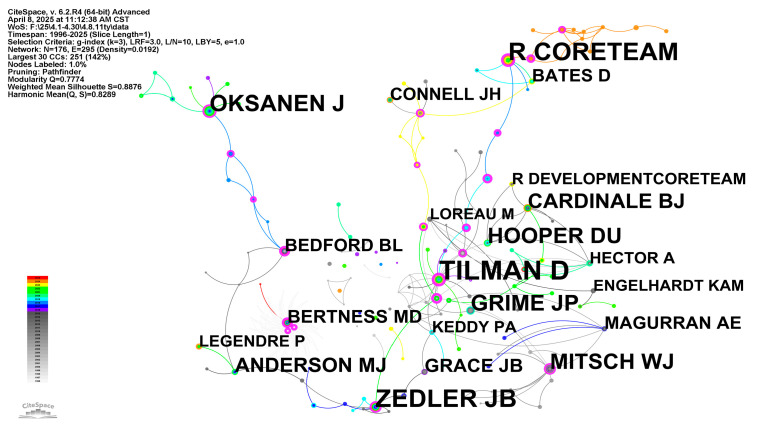
Author co-citation map of wetland plant diversity research.

The author co-occurrence map (Figure 4) and author co-citation cluster map (Figure 5) respectively characterize the structure and evolution of scholarly collaboration networks and academic influence networks. Analysis of these dual mappings reveals collaborative patterns among research groups, core members of academic communities, and knowledge diffusion trajectories—all critical for understanding a field’s scholarly ecosystem [55]. Figure 4 displays the structural distribution of multiple collaborative groups or academic communities across temporal periods. Nodes with intense circumferential coloring denote either periods of active collaboration or pivotal transitional roles in the network’s evolution. The most prominent node, “Alberti, Juan” (rendered in maximal font size at the network’s topological core), demonstrates exceptional collaboration frequency and betweenness centrality, suggesting either disciplinary leadership or institutional gatekeeping. His polychromatic time-slice rings further indicate sustained research activity in wetland phytodiversity. His scholarly contributions primarily focus on zoogenic impacts on wetland phytodiversity [56,57]. Concurrently, multiple chromatically-distinct collaborative clusters emerged around Alberti, Juan, exemplified by the 2023 academic collective centered on Cao, Xiaoai; Zhuo, Yi; and Xu, Zhichao; and the pre-2015 active collaboration nexus comprising Chang, Jie; Zhang, Chong-Bang; and Zhu, Si-Xi-collectively demonstrating transregional or transinstitutional cooperation patterns.

In contrast, the author co-citation cluster map emphasizes scholarly impact rather than collaboration. Node size corresponds to citation frequency, while edge density indicates co-citation strength. Structurally, the map reveals a dense co-citation network centered on “Tilman, D”, surrounded by tightly clustered highly-cited authors (“Grime, J.P.”; “Hooper, D.U.”; “Loreau, M.”; “Mitsch, W.J.”) forming the field’s theoretical foundation. Prominent peripheral clusters include “Oksanen, J.”, “R Core Team”, among others, associated with the R programming language and its ecological statistical packages (e.g., vegan), demonstrating the field’s methodological emphasis [16,58]. Among them, Tilman, D. has demonstrated a wide range of research interests in the field of plant ecology, such as the relationship between plant diversity and ecosystem function [59]; the impact of human activities on plant diversity [60] and the prediction of models on ecological community construction [61]. Additionally, authors including “Connell, J.H.”, “Bates, D.”, and “Legendre, P.”, among others, constitute secondary hubs, reflecting the disciplinary knowledge structure’s diversity and stratification.

Comparative analysis reveals the co-occurrence network exhibits stronger geographical signatures, reflecting collaborative sociology and strategic alliances, whereas the co-citation network manifests intellectual lineage transmission and knowledge diffusion, revealing hierarchical knowledge-influence structures. Authors like ‘Zedler, J.B.’ are active in both networks, serving as key nodes in collaboration networks and important contributors in knowledge structures. These “dual-core” authors often become central forces driving disciplinary development. Synthetically, author co-occurrence and co-citation maps jointly reveal the organizational structure, key figures, core literature, and knowledge evolution paths of academic groups in a research field. Through this multi-dimensional scientometric mapping analysis, we can not only grasp the current academic ecology, but also provide strategic guidance for future research collaboration and literature selection.

### 3.5. Countries and Institutions

**Figure 6:**
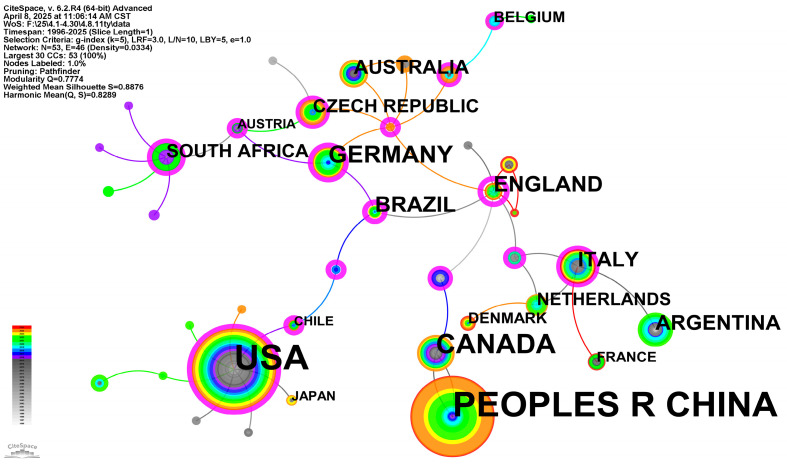
Country co-occurrence map of wetland plant diversity research.

CiteSpace-based analysis of the country/region co-occurrence map of wetland plant diversity research (Figure 6) reveals the global research pattern of the field and its major collaborative networks. Node size directly correlates with national publication output in this field, where the United States, China, Australia, Germany, the UK, and Canada dominate with largest nodes, signifying their status as primary contributors to wetland plant diversity research. Purple outer rings denote betweenness centrality values, indicating a nation’s brokerage role in international collaboration networks. The US, UK, Germany, Australia, and Canada exhibit elevated betweenness centrality, demonstrating that they not only produce a large number of research outputs themselves, but also play a key role in connecting different countries or research groups for collaboration. Inter-country links represent co-occurrence relationships, typically indicating collaborative publications between researchers in these countries, with line thickness potentially signifying collaboration intensity. The map shows dense international collaboration clusters including North America (US–Canada), intra-European networks (Germany–UK–Netherlands), and Europe–Australia–China linkages.

Additionally, colored edges in the map represent citations or collaborations from different time periods. Analyzing color evolution trends allows deeper investigation of thematic progression and emerging research fronts. From the perspective of the time series of node color, the main scientific research output countries such as the United States, Germany, and the United Kingdom show long-term research accumulation, and the node color covers the earlier and more recent time periods, while countries like China with warm node color (representing the recent one) show a significant increase in the activity and influence of scientific research in this field in recent years. Overall, the national co-occurrence network outlines a global research landscape with a small number of high-yielding and highly centralized countries at its core, supplemented by extensive international cooperation links, reflecting that wetland plant diversity research as a global issue requires cross-border knowledge exchange and collaboration.

**Figure 7:**
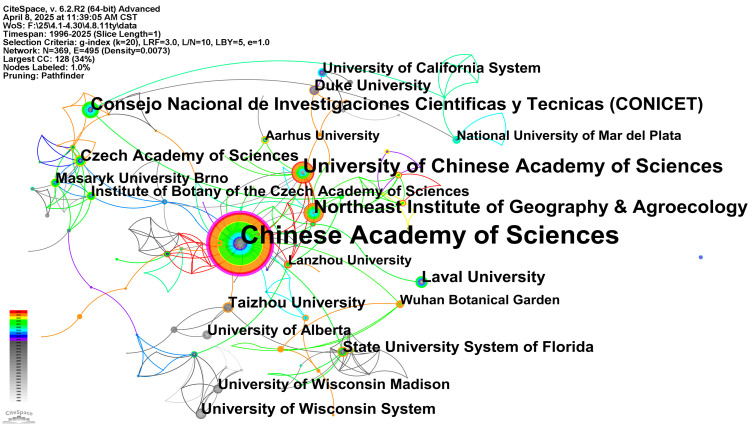
Institutions co-occurrence map of wetland plant diversity research.

Figure 7 displays core research institutions and their global collaboration networks in this field. Node size directly correlates with institutional publication count. The Chinese Academy of Sciences (CAS) occupies the central position with its substantially larger node, which is significantly higher than other institutions, demonstrating global leadership in research output. The Purple node borders indicate betweenness centrality of the institution in the cooperative network. CAS’s exceptionally high betweenness centrality signifies its critical bridging role in connecting diverse institutions and facilitating international collaborations. Several university systems and research universities in the United States, such as the University of California System, Duke University, and the University of Wisconsin System, University of Wisconsin Madison, have also shown to be larger nodes and higher intermediary centrality, and are important contributors to the field and key nodes in collaborative networks.

Connecting lines between institutions represent collaborative relationships in co-authored papers. The density and distribution of these lines reveal cooperation patterns among different institutions. Close cooperation within countries (e.g., between universities in the United States) can be observed, as well as significant cross-border cooperation links, such as between the Chinese Academy of Sciences and institutions in Australia, Germany, the United States, etc., as well as between different institutions in Europe. From the perspective of the temporal distribution of node color, the node color of major institutions such as the Chinese Academy of Sciences covers a wide time range, indicating that it has a long-term research foundation in this field and has maintained a strong development momentum in recent years. The color distribution of nodes in some other important institutions also reflects their different research accumulation and active periods. Overall, the Institutional Co-Occurrence Network clearly depicts a global research pattern of wetland plant diversity with the Chinese Academy of Sciences as the core, and many prolific and highly connected research institutions in the United States, Europe and other regions as the skeleton, building an extensive international cooperation network.

### 3.6. Keywords

**Figure 8:**
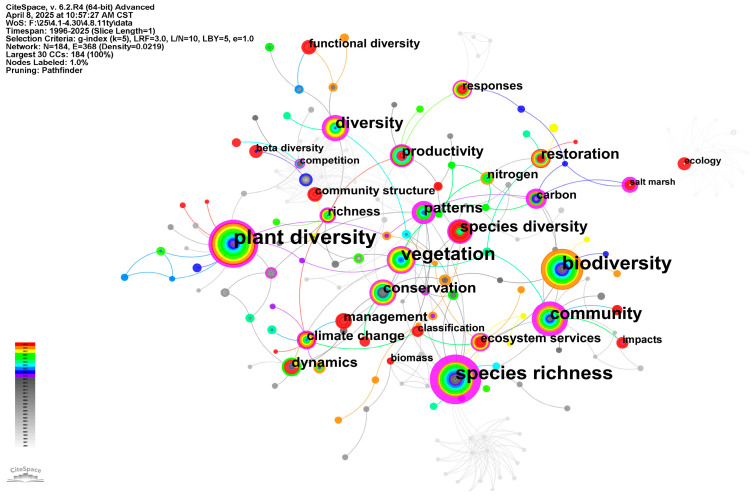
Keyword co-occurrence map of wetland plant diversity research.

Figure 8 elucidates research hotspots and intellectual architecture in wetland phytodiversity studies through keyword co-occurrence network analysis. The size of nodes in the graph is positively correlated with the frequency of keywords in the literature, and the core terms such as “plant diversity”, “species richness”, “biodiversity”, “vegetation”, and “community” have large nodes, highlighting their status as the most basic and popular research topics in this field. The purple outer circle of the node indicates its intermediary centrality, reflecting the role of keywords as a bridge in connecting different research topic networks. “Plant diversity” occupies the network epicenter with maximal intermediary centrality, and is a key hub to connect other concepts in the field. Similarly, high-frequency keywords such as “species richness”, ”biodiversity”, ”vegetation”, ”community”, ”conservation”, and “management” also show high intermediary centrality, indicating that these concepts are not only important research objects, but also key nodes for integrating different research perspectives and methods. This shows that researchers’ research on wetland plant diversity is multidimensional, based on the current status of plant diversity and species richness, with further focus on diversity conservation and ecosystem restoration and management strategies [62].

The connecting lines between keywords denote their co-occurrence frequency in the scholarly literature, with thicker or denser lines indicating stronger conceptual linkages that collectively define specific research frontiers. The knowledge graph demonstrates that research themes radiate from the “plant diversity” nucleus along several dimensions:, including in-depth exploration of its composition and structure (e.g., ‘species richness’, ‘community structure’, ‘community’, ‘patterns’), its functional attributes (e.g., ‘functional diversity’, ‘productivity’, ‘biomass’, ‘ecosystem services’), and the close connection with its application and management (e.g., “conservation”, “management”, “restoration”). In addition, wetland plant diversity research has also been widely associated with environmental change factors (such as “climate change”, “nitrogen”, “carbon”, “responses”, “impacts”), showing the research’s concerns about wetland ecosystem responses in the context of global change [21]. For example, Li et al. [62] focus on the impact of climate change on the distribution pattern of plant diversity in wetlands on the Qinghai-Tibet Plateau, and determine the critical area for the protection of endemic wetland plants under future climate change scenarios. At the same time, the impact of human activities such as reservoir construction, urbanization and grazing on wetland ecosystems has also attracted widespread attention [21]. The results of a study conducted by Yuan et al. [20] on the wetlands of the Yellow River Basin in China showed that human disturbance adversely affected wetland plant diversity, but population density had a positive effect on the β diversity of wetland plants in the Yellow River. From the perspective of the temporal distribution of node colors, the node colors of the core basic concepts (such as “plant diversity”, “species richness” and “biodiversity”) span a long time period, reflecting their long-term research history; Some keywords related to global change, functional diversity, ecosystem services and restoration are connected to recent nodes, suggesting that these are emerging or continuing research frontiers in this field in recent years. In summary, the keyword co-occurrence map depicts a dynamic knowledge graph centered on the core concept of plant diversity, focusing on multiple dimensions such as composition, function, application, and environmental response, and continuously expanding and deepening the research topic over time.

The field employs a diverse methodological toolkit that has evolved to address increasingly complex questions. Foundational research relies heavily on field observation and long-term monitoring of permanent plots to track changes in community composition over time, often complemented by controlled experiments (mesocosms) to isolate the effects of specific environmental drivers like hydrology or nutrient levels [63]. A significant trend is the widespread adoption of trait-based approaches, which measure plant functional traits to mechanistically link species to ecosystem functions [64]. Concurrently, the rise of molecular techniques like eDNA metabarcoding requires sophisticated bioinformatics methods to process massive sequencing datasets for comprehensive biodiversity assessment [65]. To scale these findings, researchers increasingly use remote sensing and spatial analysis to map vegetation patterns and health across entire landscapes, while ecological modeling and network analysis are used to synthesize complex data, understand species interactions, and forecast ecosystem responses to future change [66,67].

**Figure 9:**
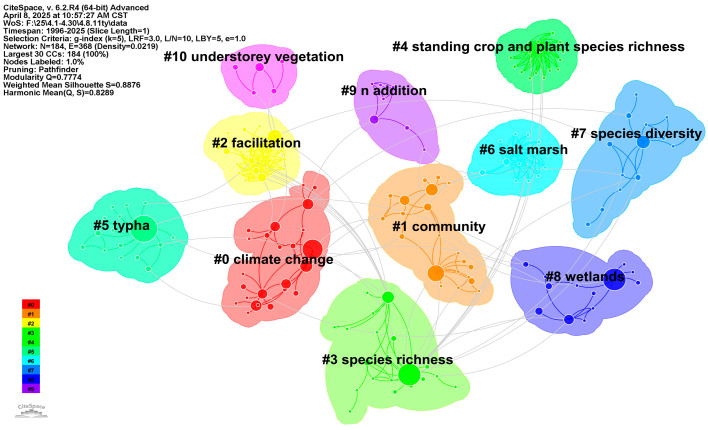
Co-occurrence keyword cluster network.

Based on the wetland plant diversity research keyword clustering map generated by CiteSpace, the main research frontiers and their interrelationships in this field are clearly presented (Figure 9). Each numbered cluster in the graph represents a relatively independent but interrelated set of research topics. The smaller the number after “#”, the more keywords there are in the cluster and the better the clustering performance [25]. As shown in Figure 9, the WOS keyword cluster includes: #0 Climate change; #1 community; #2 promotion; #3 species richness; #4 aboveground biomass and plant species richness; #5 cattails; #6 salt marsh; #7 species diversity; #8 wetlands; #9 n added; #10 underforest vegetation. These clusters can be broadly divided into four categories:

(1) Core Biodiversity and Community Ecology: “community”; “species richness”; and “species diversity”. These studies focus on the composition, structure, diversity measurement, and fundamental distribution patterns of wetland plant communities across different habitats, emphasizing key indicators like species richness and diversity as foundational research pillars. For instance, Sharpe et al. [68] used plant species richness as an indicator and observed nonlinear patterns of species richness along the Nanticoke River, which may represent typical characteristics in relatively undisturbed estuarine systems. Concurrently, recent studies by multiple scholars have utilized wetland plant community characteristics as quantitative indicators of wetland degradation [69], recognizing that understanding plant species diversity is a prerequisite for wetland restoration [70].

(2) Environmental Drivers and Global Change: “climate change” and “n addition”. Such studies show the trend of integrating global change context into ecological research, with researchers focusing extensively on the impact of climate change on wetland plant diversity and its associated responses [71], and highlighting the impact of nutrient enrichment on wetland plant diversity and its community structure [49], reflecting the closely focused attention of the field on current global environmental challenges.

(3) Specific Habitats and Taxa: “wetlands”; “salt march”; “*typha*” and “understorey vegetation”. These clusters cover research on plant diversity in the overall environment of wetlands and conduct more refined ecological research from a wide range of wetlands to specific wetland types, specific plant genus or specific vegetation levels. As a highly heterogeneous ecosystem, salt marshes’ unique hydrological processes play decisive roles in vegetation distribution patterns and the structural-functional stability of wetland ecosystems, holding significant research value [69]. Meanwhile, studies on specific species may indicate focus on important or invasive species. For example, Houlahan et al. investigated native species richness (including *Typha*) responses to exotic species richness, concluding that controlling dominant species spread is crucial for conserving inland wetland biodiversity [72].

(4) Ecological Processes and Functions/Indicators: “facilitation” and “standing crop and plant species richness”. These studies extend beyond static diversity descriptions to examine positive plant interactions, productivity-diversity relationships, and ecological processes sustaining diversity, along with biodiversity-ecosystem function or biomass linkages. Understanding plant facilitation effects on community structure and ecosystem functions enhances knowledge of community organization mechanisms, offering new approaches for degraded wetland restoration [73]. Zuidam et al. [69] evaluated multi-species introduction effects on wetland community restoration, finding that high functional diversity plant species could stimulate floating fen community recovery in the first two years.

These four themes are intertwined to form a multidimensional framework for the study of wetland plant diversity: the study starts from describing the basic characteristics of plant communities and diversity in wetlands, and deeply analyzes how environmental factors such as climate change and human activities affect these diversity; At the same time, the research also focuses on specific wetland types or plant groups, and explores ecological processes such as competition and facilitation, as well as the relationship between diversity and biomass/function. This research framework highlights the complexity, multidisciplinary intersection of wetland plant diversity research and its importance in understanding and responding to the degradation of functions and biodiversity loss of wetland ecosystems in the context of global environmental changes, providing important scientific support for scholars, managers and government agencies in managing wetland ecosystems, promoting restoration and ensuring ecological security.

**Figure 10:**
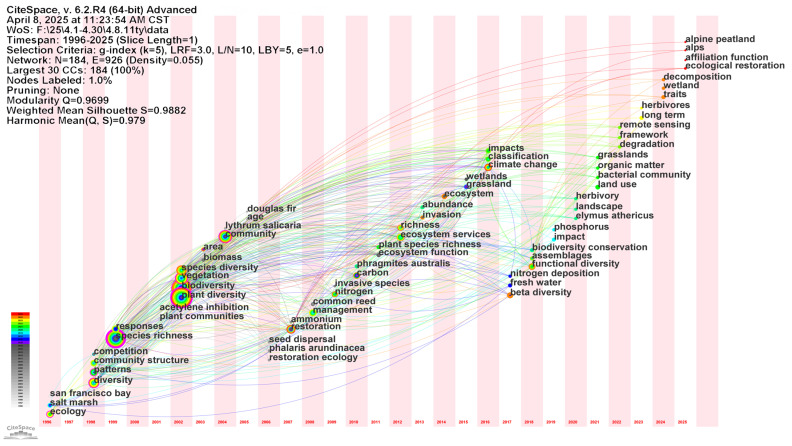
Time zone view of keywords of wetland plant diversity.

In the keyword timezone map (Figure 10), research focuses changed over different periods. The distribution of keyword nodes along the horizontal time axis in the graph reveals the emergence and active period of different concepts. Earlier studies (before 2005) focused on basic ecological descriptions of wetland plant communities [74], including species characteristics such as species richness, plant diversity, biodiversity, community structure, community and its patterns, area and other spatial patterns. At the same time, specific wetland types or regions such as salt march have attracted much attention due to their huge potential in biodiversity conservation [8], and explore some of the fundamental interactions and ecophysiological processes such as competition (e.g., acetylene inhibition).

In the middle period (2005–2015), the research topics gradually expanded, and more attention began to be paid to ecosystem functions and ecosystem services, as well as key environmental factors affecting wetland plant diversity such as nitrogen and carbon [49,75], and more attention was paid to invasive species and their management [76]. For instance, McGlynn investigated the ecological impacts of invasive *Phragmites australis Linnaeus* and *Lythrum salicaria* L. on native vegetation [77]. At the same time, the ecological function of wetlands has been paid more and more attention, and many countries have adopted policies to restore the natural state of wetlands, and restoration ecology has begun to receive attention as an applied theme [78,79].

In the recent past (after 2015), the research frontier has been further tilted towards global changes such as climate change and their “impacts, responses” on wetland ecosystems [42], the application of advanced measurement and assessment methods such as remote sensing has increased [80], and the understanding of diversity has expanded from simple species numbers to functional diversity and beta diversity. In addition, the keywords “phosphorus”, “decomposition”, “herbivory”, “traits”, “landscape”, and “biodiversity conservation” reflect the fact that the study also delves into more specific environmental factors, ecological processes, plant traits, landscape level, and bioconservation strategies and covers a wider range of wetland types, such as alpine peatland and wetlands and grasslands [81,82].

The links between the keywords clearly show the direction of the flow of knowledge, for example, from the early concept of diversity to the study of ecosystem function and invasive species, and then further to the impact of climate change and higher measures of diversity, which reflects the evolution of wetland plant diversity research from basic description to mechanism discussion, from single factor to multiple impacts, from species level to function and landscape level, and is increasingly closely integrated with global environmental issues.

### 3.7. Prospects of Wetland Plant Diversity Research

Currently, wetland plant diversity research has become a crucial branch in ecology and environmental conservation, with its research status being elucidated through bibliometric analysis. CiteSpace analysis of 482 documents reveals global research patterns, core research strengths, and thematic evolution trends in this field. The results show that the number of publications in Plant Diversity Research has been on the rise over the years, but the annual output is still relatively small (<50 papers/year), highlighting the untapped potential of the field. While basic frameworks for Plant Diversity Research exist, significant gaps remain in translating findings into ecological management practices. Based on the analysis of the current status of wetland plant diversity research and the trajectory of its thematic evolution, future research trends are expected to be deepened and expanded in several key directions:

(1) Quantifying Climate Change Vulnerability and Resilience of Wetland Plant Functional Diversity and Associated Ecosystem Services: Research should prioritize integrative studies that mechanistically link climate change impacts (e.g., altered hydrological regimes, temperature extremes, sea-level rise) to shifts in plant functional traits, subsequent changes in functional diversity, and the consequences for critical ecosystem services such as carbon sequestration, water quality regulation, and habitat provision [83]. At the same time, a deeper understanding of the mechanism of wetland plant diversity under the combined effects of climate change and human activities will be the core of future research [21]. This moves beyond documenting patterns to fostering predictive understanding crucial for developing effective climate adaptation strategies for wetlands.

(2) Developing and Implementing Integrated, AI-Driven Monitoring Systems for Dynamic Wetland Assessment using Multi-Source Remote Sensing and eDNA Metabarcoding: Future efforts should focus on the strategic fusion of advanced remote sensing techniques (capturing habitat structure, extent, and condition) with eDNA metabarcoding (for rapid, comprehensive biodiversity detection, including rare, invasive, or cryptic species) [84,85]. Leveraging AI and machine learning for the integration and analysis of these diverse data streams can lead to the development of scalable, cost-effective, and near real-time monitoring and early warning systems for wetland degradation and biodiversity change [86].

(3) Elucidating the Role of Belowground Plant Diversity and Biogeochemical Processes in Wetland Carbon Sequestration and Nutrient Cycling under Global Change: While much research focuses on aboveground components, belowground dynamics (root systems, microbial communities, soil organic matter formation) are paramount for long-term carbon storage (especially relevant for blue carbon ecosystems and peatlands) and nutrient cycling [87]. Future research should investigate how wetland plant diversity, including belowground traits, influences these critical soil processes and how these interactions are affected by climate change, nutrient enrichment, and other anthropogenic stressors [88].

(4) Advancing Socio-Ecological Systems (SES) Research to Enhance the Effectiveness, Equity, and Sustainability of Wetland Restoration and Wise Use: The long-term success of wetland conservation and restoration is inextricably linked to human behavior, governance structures, and socio-economic contexts [89]. An SES approach, integrating ecological knowledge with social science perspectives, is needed to analyze the complex feedbacks between human and natural systems in wetlands. Future research should evaluate the long-term ecological and social outcomes of restoration projects, assess the role of local communities and indigenous knowledge in wetland management, and develop adaptive governance models that promote both ecological health and equitable human well-being [90].

Overall, research must strengthen the integration of fundamental theories with practical applications, accelerating the translation of scientific findings into wetland protection policies, management protocols, and restoration practices to address global wetland challenges.

### 3.8. Limitations

This study investigates wetland plant diversity research status and trends using bibliometric methods, yet has several limitations: (1) The data primarily sourced from Web of Science Core Collection, while containing high-quality journals, may incompletely cover relevant publications, especially regional studies or non-SCI/SSCI indexed literature, potentially introducing dataset bias. (2) Non-English databases (e.g., China National Knowledge Infrastructure [CNKI], Korean database [Nurimedia, Seoul, Republic of Korea]) and their publications were excluded. (3) Although Bibliometric analysis software such as CiteSpace is powerful, its clustering, centrality calculation and emergence-detection results are affected by parameter settings, and the analysis results mainly reflect quantitative characteristics such as the number of documents and citation relationships, which cannot directly evaluate the quality and scientific contribution of the research content, and the understanding of the research topic still needs to be combined with the in-depth interpretation of the core literature content. (4) Bibliometric analysis fundamentally delineates macro-level trends and knowledge structures from existing publications, with identified research fronts and hotspots being historical data-derived inferences that cannot precisely predict actual future research directions.

## 4. Conclusions

The bibliometric analysis of wetland plant diversity research from 1986 to 2025 reveals a field characterized by significant growth and dynamic evolution. This evolution can be broadly categorized into three phases: an initial period of Nascent Exploration (1986–2006, Perth, WA, Australia) with limited publications focusing on foundational ecological factors; a phase of Methodological Expansion (2007–2019) marked by an increase in publications driven by advancements in survey techniques like remote sensing and molecular ecology, and an intensified focus on ecosystem services; and a recent phase of Technology Convergence (2020–2025), where the field has achieved maturity with high growth, integrating transdisciplinary approaches and addressing urgent global challenges like climate change and biodiversity loss. Research output is dominated by countries such as the United States, China, Australia, Germany, the UK, and Canada, with institutions like the Chinese Academy of Sciences playing a central role in a robust international collaboration network. Thematic evolution shows a clear trajectory from fundamental studies on species composition and diversity metrics towards more complex and applied research on ecosystem functions and services, the impacts of climate change, functional diversity, wetland restoration, and the increasing application of sophisticated technologies. This dynamism demonstrates a field actively adapting to provide scientific understanding for increasingly complex and pressing environmental concerns.

The strong research focus on climate change impacts, biodiversity loss, and ecosystem services directly underscores the urgent need for strengthening national and international wetland conservation policies, aligning with global frameworks such as the Ramsar Convention’s Fifth Strategic Plan (2025–2034) and the Kunming-Montreal Global Biodiversity Framework. The identification of leading research countries and institutions can inform strategic international collaborations to address transboundary wetland issues and build capacity in regions with limited research infrastructure. Furthermore, research on ecosystem functions, restoration ecology, and the impacts of human activities provides crucial scientific underpinning for developing adaptive management strategies and implementing more effective, evidence-based wetland restoration projects. The call to “harness wetlands as solutions” for challenges like pollution reduction and climate resilience is directly supported by the growing body of research on wetland ecosystem services, a key theme identified in the bibliometric analysis. This analysis can therefore serve as a valuable resource for policymakers, highlighting areas of strong scientific consensus, identifying critical knowledge gaps that require further research to inform specific policy questions (e.g., quantifying blue carbon for national climate commitments), and pinpointing key scientific partners for knowledge co-production and dissemination.

To address pressing knowledge gaps and capitalize on emerging opportunities, future research should prioritize several specific, evidence-based directions. Firstly, quantifying the vulnerability and resilience of wetland plant functional diversity and associated ecosystem services to climate change by mechanistically linking impacts to functional trait shifts and service provision. Secondly, developing and implementing integrated, AI-driven monitoring systems that fuse multi-source remote sensing data with eDNA metabarcoding for dynamic, scalable, and near real-time wetland assessment. Thirdly, elucidating the role of belowground plant diversity and biogeochemical processes in wetland carbon sequestration and nutrient cycling under global change, particularly focusing on soil dynamics and their response to stressors. Finally, advancing Socio-Ecological Systems (SES) research to enhance the effectiveness, equity, and sustainability of wetland restoration and wise use by integrating ecological knowledge with social science perspectives to analyze human-natural system feedback and inform governance.

## Figures and Tables

**Table 1:** Top 10 ranking of most-cited of wetland plant diversity articles.

Title	Year	Total Citations	Average Number of Citations Per Year	Reference
WETLAND RESOURCES: Status, Trends, Ecosystem Services, and Restorability	2005	1479	73.95	[4]
Recent assembly of the Cerrado, a neotropical plant diversity hotspot, by in situ evolution of adaptations to fire	2009	774	48.38	[47]
Temperate freshwater wetlands: types, status, and threats	2002	414	18.00	[48]
Patterns in Nutrient Availability and Plant Diversity of Temperate North American Wetlands	1999	405	15.58	[49]
Plant Species Richness in Riparian Wetlands—a Test of Biodiversity Theory	1998	388	14.37	[50]
Effects of macrophyte species richness on wetland ecosystem functioning and services	2001	328	13.76	[34]
Wetlands as large-scale nature-based solutions: Status and challenges for research, engineering and management	2017	222	27.75	[51]
Core Microbiota in Agricultural Soils and Their Potential Associations with Nutrient Cycling	2019	178	29.67	[52]
The effects of adjacent land use on wetland species richness and community composition	2006	164	8.63	[53]
Patterns of fungal diversity and composition along a salinity gradient	2011	152	10.86	[54]

## Data Availability

The original contributions presented in this study are included in the article; further inquiries can be directed to the corresponding author.
